# Outcomes of patients presenting with Guillain-Barre Syndrome at a tertiary care center in India

**DOI:** 10.1186/s12883-022-02676-4

**Published:** 2022-04-22

**Authors:** Priyank Patel, Darshil Shah, Chinmay Jani, Jui Shah, Ruchi Jani, Arjun Kelaiya, Jinal Pandya, Harpreet Singh, Omar Al Omari, Dhara Roy, Irmgard Behlau, Ami Parikh

**Affiliations:** 1 Department of Medicine, Smt. NHL Municipal Medical College, Ahmedabad, Gujarat India; 2 Department of Internal Medicine, Temple University Hospital, Philadelphia, PA 19140 USA; 3 Harvard Medical School, Boston, MA USA; 4 Division of Medicine, Mount Auburn Hospital, Beth-Israel Lahey Health, Cambridge, MA USA; 5 Medical Student, Smt. NHL Municipal Medical College, Ahmedabad, Gujarat India; 6 GCS Medical College, Hospital & Research Centre, Ahmedabad, Gujarat India; 7 Department of Physiology, Dr. M K Shah Medical College, Ahmedabad, Gujarat India; 8 Department of Pulmonary and Critical Care, Medical College of Wisconsin, Milwaukee, WI USA; 9 Department of Infectious Disease, Mount Auburn Hospital, Beth-Israel Lahey Health, Cambridge, MA USA; 10 Molecular Biology & Microbiology, Tufts Graduate School of Biomedical Sciences, Tufts University School of Medicine, Boston, MA USA

**Keywords:** Guillain-Barre-Syndrome, Demyelination, Hughes Scale, India

## Abstract

**Background:**

The Guillain-Barre Syndrome (GBS), also known as acute idiopathic polyneuritis, is a critical acquired condition associated with preceding nonspecific infection or triggering factors like trauma, surgery, or vaccination. GBS is currently the most frequent cause of acute flaccid paralysis in India. This study evaluates the short-term and in-hospital outcomes in different subtypes of GBS.

**Methods:**

A prospective observational study was conducted at V.S. Hospital, Ahmedabad, from September 2015 to December 2017. Patients above the age of 12 were included. Patients having other underlying neurological conditions, as well as immunodeficiency disorders, were excluded. The patients were classified into different subtypes of GBS, and functional outcomes were recorded on admission and discharge according to Hughes Scoring System. All statistical analyses were performed by using SPSS software.

**Results:**

Out of 50 patients, 35 (70%) were males. The mean age was of 37.18 +/− 18.35 years. 25 (50%) patients had a preceding infection. 88% of patients presented with cranial nerve (CN) involvement had a Hughes Score of >/= 3 (*p* = 0.0087). They had less improvement of Hughes Score on discharge (0.13 +/− 0.04) as compared to the patients without cranial nerve involvement (0.38 +/− 0.08) (*p* = 0.008). Respiratory involvement was associated with a higher Hughes Score (*p* = 0.005) on admission. 85% of patients diagnosed with an axonal subtype of GBS had a Hughes Score of >/= 3 (*p* = 0.06) compared to 74% patients with demyelinating subtype. Axonal subtype required double period (11 +/− 2.34) to show improvement as compared to demyelinating subtype (6 +/− 1.2) (*p* = 0.020). Irrespective of the subtypes, in two different treatment cohorts (PLEX vs IVIG), there was no difference in short term functional outcomes measured by improvement in the Hughes scores (*p* = 0.89).

**Conclusions:**

Early cranial nerve and respiratory involvement in patients presenting with GBS are associated with poor outcomes warranting immediate critical care involvement. In our study, amongst all the subtypes, axonal had poor clinical outcomes. Further clinical trials on the Indian subpopulation will help us evaluate the impact of different treatment modalities on this disease.

## Introduction

The Guillain- Barre Syndrome (GBS) is an acquired autoimmune condition involving the peripheral nervous system. It is also known as post-infectious polyneuropathy or acute idiopathic polyneuritis. It often leads to respiratory or bulbar compromise. GBS can have variable presentations, including gait disturbance, pain, weakness, rapidly ascending symmetric flaccid muscle paralysis, areflexia with a distal predominance (involving lower motor neurons), sensory disturbance, and variable autonomic involvement, increased cerebrospinal fluid protein without pleocytosis [1].

Incidence rate of GBS is 1–2 per 100,000 population [2, 3]. The likelihood of an individual acquiring GBS in a lifetime is 1:1000 [4]. Classification of GBS into different groups of syndromes is based on clinical features and electrodiagnostic criteria [5]. The most frequent cause of acute flaccid paralysis in India is GBS, which constitutes a serious neurological emergency, affecting all ages, including children, with male predominance. Preceding nonspecific infection or triggering factors like trauma, surgery, or vaccination, are frequently associated with GBS, usually a few days to weeks before neurological symptoms. Overall, younger patients with GBS have a more favorable course and prognosis [5].

Most studies attempted to identify functional outcomes in GBS associated with prognostic factors such as age, rate of progression, and need for respiratory support. However, most of the studies had a retrospective design, and only a few were prospective [6, 7].

In India, the incidence of acute inflammatory demyelinating polyneuropathy (AIDP) and acute motor axonal neuropathy (AMAN) is equal [8]. AMAN is more common in younger patients [8]. AIDP is the most common form of GBS worldwide [9]. In AIDP, the immune attack is directed at peripheral nerve myelin [9]. On the other hand, AMAN is characterized by decreased compound muscle action potentials (CMAP) and the absence of demyelinating findings in electrophysiological studies [10]. Different treatments have been assessed based on the functional ability of patients at presentation, during, and post-hospitalization [11]. However, the functional ability of a patient is rarely used as a prognostic factor to predict the patient’s functional outcome, need for Intensive Care Unit (ICU) admission, or length of hospital stay [4, 12]. There is limited data regarding functional outcomes in GBS [13].

In this study, we prospectively evaluated the clinical outcome of patients of > 12 years of age with different subtypes of GBS and their functional outcomes. We measured the following functional outcomes: antecedent events, cranial nerve involvement, treatment, duration of hospital stays, and complications. Along with cerebrospinal fluid (CSF) findings and electrophysiological investigations, we used the Hughes Scale to assess factors used to determine prognosis in GBS.

## Methods and materials

This was a prospective observational study conducted at Sheth V.S. Hospital, a tertiary care hospital in Ahmedabad, Gujarat, between September 2015 and December 2017. We collected data from 50 patients that were diagnosed with GBS. Our inclusion and exclusion criteria are given below.

### Inclusion criteria

1. Fulfill diagnostic criteria for GBS by the National Institute of Neurological Disorders and Stroke.
2. Patients with Miller Fisher Syndrome (MFS) and all other variants of GBS, including overlap syndromes, can be included.
3. All males and females of > 12 years, independent of disease severity.
4. Presented within 2 weeks of the onset of symptoms.

### Exclusion criteria

1. Pregnancy.
2. Known severe allergic reaction to correctly matched blood products.
3. Known selective IgA deficiency, HIV reactive, and hypokalemia.
4. Previous steroid therapy.
5. Severe concurrent disease.

#### Clinical evaluation, variable definition, and investigations

We collected detailed history related to the onset of symptoms, duration, and progression. In addition, antecedent history, including fever, diarrhea, upper respiratory tract infection, vaccination, history of diabetes, or other illnesses, was collected. A detailed general examination includes vitals, oxygen saturation, and single breath count in all patients, and systemic review, including the respiratory system, cardiovascular system, and neurological examination. Respiratory failure was judged based on the standard criteria: use of accessory muscles; inability to speak in full sentences; severe tachypnea; paradoxical abdominal muscle activity. Clinical judgement was augmented by measuring the forced vital capacity, negative inspiratory force, and maximum expiratory pressure periodically where applicable.

A complete blood workup and electrophysiological studies were done in all patients. In addition, we performed antinuclear antibodies, antiganglioside antibody panel, urine porphyrin, and imaging of the spine in selected patients. We classified GBS based on Modified Rajabally’s electrophysiologic subtype [14] as AIDP, AMAN, and acute motor and sensory axonal neuropathy (AMSAN). In all patients, the functional disability was scored according to the HUGHES Scale (Table 1) [15]. We recorded this score prospectively also on discharge. The baseline characteristics were reported as the mean (standard deviation) and percentage for quantitative and categorical variables, respectively. Different statistical tests, including chi-square (nominal variables), t-test, and Mann-Whitney U test (for continuous), were conducted to evaluate the significance of difference. We used SPSS version 21 (SPSS, Chicago, IL) and SAS version 9.1 for all statistical analyses.

**Table 1:** Guillain Barré Syndrome disability scale, adapted from Hughes et al.

Score	Clinical examination
0	A healthy state
1	Minor signs and symptoms
2	Able to walk for 10 m or more without support
3	Able to walk 10 m with support
4	Confined to bedn
5	Required assisted ventilation
6	Dead

#### Treatment and follow-up

All the patients diagnosed with GBS were treated with plasma exchange (PLEX) or intravenous immunoglobulins (IVIG), irrespective of the subtype of GBS. A total of 50% of patients were treated with PLEX, 32% were treated with IVIG, and no treatment was given to the remaining 18% of the patients. All the patients were admitted and were closely observed throughout their hospital stay. According to the Hughes Scale on admission and discharge, all the patients were graded to obtain the functional outcomes.

## Results

A total of 50 patients were diagnosed with GBS and later classified into various subtypes.

### Age and gender distribution

Most of the patients were in the second decade (26%) and fifth (22%) decade. The mean age was (37.18 ± 18.35). 35 (70%) were male, and 15 (30%) were female. The male-to-female ratio is 2.3:1.

### Antecedent infections

**Fig. 1:**
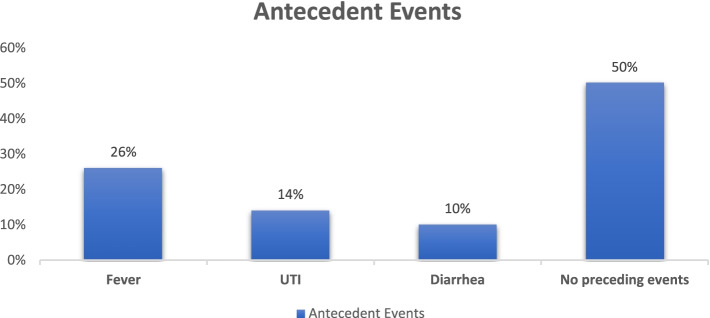
Antecedent events in patients with GBS in our study (total, *n* = 50)

50% of patients had preceding infections. In our study, on admission, 88% of patients with an antecedent event (*n* = 25) had a higher Hughes score ≥ 3 as compared to 72% patients without identifiable antecedent event (*n* = 25) with Hughes score ≥ 3. (*p* = 0.099) (Fig. 1).

#### GBS variants

Classic GBS (*n* = 47) was much more common than atypical variants. 2 cases of paraparesis and one case of bifacial weakness with paresthesia were present.

#### Cranial nerve involvement

**Fig. 2:**
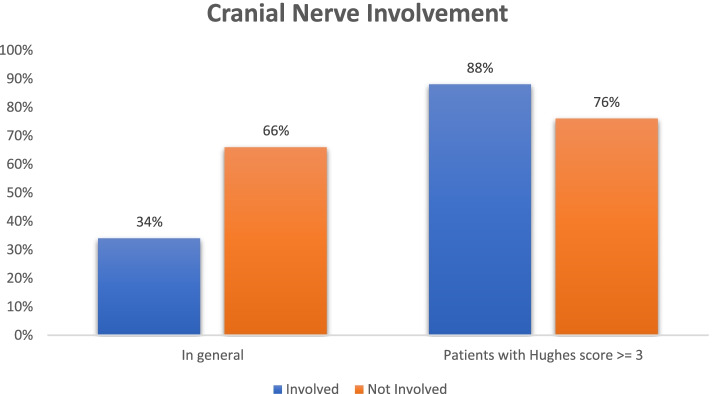
Cranial Nerve Involvement

34% of the patient had one or the other cranial nerve involvement. The facial nerve was the most common (28%) cranial nerve involved in our study. Isolated facial nerve weakness was present in 18%, and isolated bulbar weakness was present in about 6% of all the patients with cranial nerve involvement. In our study, 88% of patients with cranial nerve involvement presented with a Hughes Score ≥ 3, (*p* = 0.0087) in comparison to 76% of patients without cranial nerve involvement. The mean improvement in Hughes score upon discharge was significantly less, 0.13 ± 0.04 in patients with cranial nerve involvement (*n* = 17) as compared to 0.38 ± 0.08 in patients without cranial nerves involvement (*p* = 0.008). Meantime to improve power grade by 1 in patients with bulbar and/or facial weakness was 12 ± 2.47 days compared to 7 ± 1.86 days in patients without cranial nerves involvement (*p* < 0.001) (Fig. 2).

#### Electrophysiological types

In our study, 85% of patients with axonal subtype (*n* = 20) had Hughes Score ≥ 3 as compared to 74% patients with demyelinating subtype (*n* = 27) with Hughes Score ≥ 3 (*p* = 0.06). Improvement in score on discharge was 0.3 ± 0.11 in patients with axonal subtype as compared to 0.55 ± 0.15 improvement in score in demyelinating subtype (*p* = 0.078). Axonal neuropathy (AMAN = 11 ± 2.34 days, AMSAN = 10 ± 1.0 days) patients took almost double-time as compared to demyelinating neuropathy (AIDP = 6 ± 1.2 days) patients in improving power by one grade (*p* = 0.020) (Table 2). We used Modified Rajabally’s criteria for classifying GBS electrophysiologically [14]. Rajabally’s criteria also have an equivocal subtype. None of the patients fell into that subtype.

**Table 2:** Classification based on electrophysiological studies

AIDP	AMAN	AMSAN	Normal	Total
27 (54%)	16 (32%)	4 (8%)	3 (6%)	50

#### Sensory complaints

25 (50%) patients had sensory complaints such as tingling and numbness, and 2 (4%) patients had autonomic features such as tachycardia and postural hypotension. Out of 25, 22 (82%) patients had demyelinating, and 3 (15%) patients had axonal neuropathy.

#### Albuminocytological dissociation

CSF protein was elevated in more than half of the patients, 31 (62%) out of 50. Normal CSF was found in15 (30%) of patients, and CSF pleocytosis was there in 4 (8%) patients. Out of the 31 patients with albuminocytological dissociation, most patients had demyelination on nerve conduction (*n* = 20, 74.07%) (*p* = 0.07), and 11 had an axonal subtype of GBS on nerve conduction.

In our study, 81% of patients with albuminocytological dissociation (*n* = 31) had Hughes Score ≥ 3 on admission as compared to 79% patients without albuminocytological dissociation (*n* = 19) with Hughes Score ≥ 3 (*p* = 0.41).

Improvement in score on discharge was 0.43 ± 0.12 in patients with albuminocytological dissociation compared to 0.38 ± 0.06 improvement in score in patients without albuminocytological dissociation (*p* = 0.768).

#### Treatment given

**Fig. 3:**
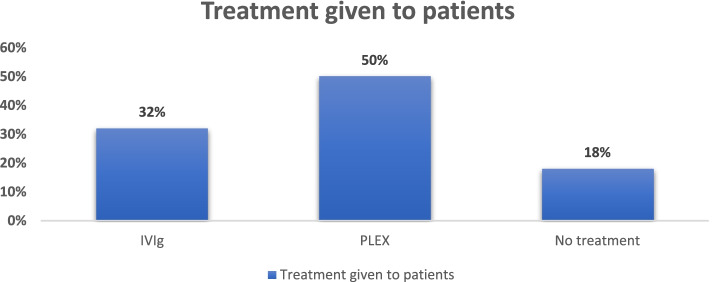
Treatment modalities for our study population

50% of the patients received PLEX (*n* = 22), about 32% of patients were treated with IVIG. In our study, the improvement in Hughes Score on discharge was 0.64 ± 0.23 in patients treated with PLEX (*n* = 25) compared to a 0.69 ± 0.04 improvement in score in patients treated with IVIG (*n* = 16). (*p* = 0.89) (Fig. 3).

We chose patients who did not receive based on their severity. At presentation, there were 10 patients with “mild GBS,” i.e. Grade 1 and 2 on Hughes Scale. These patients did not receive treatment. Out of these 10 patients, 1 deteriorated to Grade 3 on the Hughes scale, and this patient received PLEX. Therefore, 9 patients (18%) did not receive any treatment. Out of these patients, 3 improved clinically, and 6 had no change in their functional status.

#### Hughes disability scale

The maximum number of admitted patients had Hughes Scale 4 (*n* = 19, 38%), 22% of patients (*n* = 11) were in Hughes scale 3. 1 patient died. (Table 3) Improvement in Hughes score on discharge was 0.64 ± 0.23 in patients treated with PLEX (*n* = 25) as compared to 0.69 ± 0.04 improvement in score in patients treated with IVIG (*n* = 16). (*p* = 0.89) (Tables 4 and 5).

**Table 3:** Hughes score on admission and discharge

Hughes Disability Score	On admission (number of patients, %)	On discharge (number of patients, %)
1	2 (4%)	3 (6%)
2	8 (16%)	13 (26%)
3	11 (22%)	14 (28%)
4	19 (38%)	19 (38%)
5	10 (20%)	0
6	0	1 (2%)
Mean	3.56 ± 1.14	3 ± 0.95

**Table 4:** Hughes score on admission

	Bad (3,4,5) (***n*** = 40)	Good (1,2) (***n*** = 10)	***P*** value
Age	43.15 ± 20.04	37.19 ± 19.46	
Male(*n* = 35)	28 (80%)	7 (20%)	NS
Female(*n* = 15)	12 (80%)	3 (20%)	
Antecedent Events (*n* = 25)	22 (88%)	3 (12%)	0.099
Without Antecedent Events (*n* = 25)	18 (72%)	7 (28%)	
Sensory Symptoms (*n* = 25)	21 (84%)	4 (16%)	0.12
Without Sensory Symptoms(*n* = 25)	19 (76%)	6 (24%)	
Cranial Nerve Involvement (*n* = 17)	15 (88.23%)	2 (11.76%)	***0.0087***
Without Cranial Nerve Involvement(*n* = 33)	25 (75.75%)	8 (24.26%)	
Respiratory Involvement (*n* = 15)	14 (93.33)	1 (6.67%)	***0.005***
Without Respiratory Involvement(*n* = 35)	26 (74.28%)	9 (25.72%)	
Albuminocytological Dissociation (*n* = 31)	25 (80.64%)	6 (19.36%)	0.41
Without ACD(*n* = 19)	15 (78.95%	4 (21.05%)	
Subtype: Demyelinating(*n* = 27)	20 (74.07%)	7 (25.93%)	0.06
Axonal(*n* = 20)	17 (85%)	3 (15%)	
Ventilatory Support(*n* = 10)	9 (90%)	1 (10%)	***0.01***
Without Ventilatory Support(*n* = 40)	31 (77.5%)	9 (22.5%)

**Table 5:** Hughes score on admission and discharge

	Mean Hughes score on admission	Mean Hughes score on discharge	Improvement in scale	***P-***value
**Antecedent events**				
With (*n* = 25)	3.96 ± 1.1	3.64 ± 1.0	0.32 ± 0.1	0.24
Without (*n* = 25)	3.55 ± 0.9	3.15 ± 0.81	0.4 ± 0.09	
**Cranial nerve involvement**				
With (*n* = 17)	4.23 ± 1.12	4.1 ± 1.08	0.13 ± 0.04	0.008
Without (*n* = 33)	3.6 ± 0.99	3.22 ± 0.91	0.38 ± 0.08	
**Respiratory involvement**				
With (*n* = 15)	4.04 ± 1.01	3.89 ± 0.96	0.15 ± 0.05	0.005
Without (*n* = 35)	3.52 ± 0.97	3.22 ± 0.88	0.3 ± 0.09	
**Albumino-cytologic dissociation**				
With (*n* = 31)	3.86 ± 1.09	3.43 ± 0.97	0.43 ± 0.12	0.768
Without (*n* = 19)	3.65 ± 0.98	3.27 ± 0.92	0.38 ± 0.06	
**NCS**				
Demyelinating (*n* = 27)	3.87 ± 1.2	3.32 ± 1.05	0.55 ± 0.15	0.078
Axonal (*n* = 20)	3.94 ± 1.2	3.64 ± 1.09	0.3 ± 0.11	
**Ventilatory support**				
With (*n* = 10)	4.3 ± 1.21	4.2 ± 1.20	0.09 ± 0.01	0.007
Without (*n* = 40)	3.51 ± 0.96	3.1 ± 0.82	0.41 ± 0.14	
**Treatment**				
Plasma Exchange	3.94 ± 0.99	3.3 ± 0.76	0.64 ± 0.23	0.89
IVIG	4 ± 0.81	3.31 ± 0.77	0.69 ± 0.04	

#### The total duration of hospital stays according to the type of GBS

The average stay for demyelinating neuropathy was 15 days compared to axonal neuropathy, which was 18 days (*p* = 0.03). Patient’s in PLEX cohort (*n* = 25) had a more extended hospital stay (22.8 ± 3.1) days as compared to the IVIG cohort (*n* = 16) (15.3 ± 2.1) days (*p* = 0.04).

#### Mean time to improve power by one grade depending on the Subtype pattern

Axonal neuropathy took almost double the time compared to demyelinating neuropathy in improving power by one grade on Hughes Scale. (*p* = 0.020).

**Table:** 

Subtype Pattern	Mean time to improve power by grade 1 (in days)
AIDP	6 ± 1.2
AMAN	11 ± 2.34
AMSAN	10 ± 2.1
Normal	5.5 ± 1.0

#### Complications

**Fig. 4:**
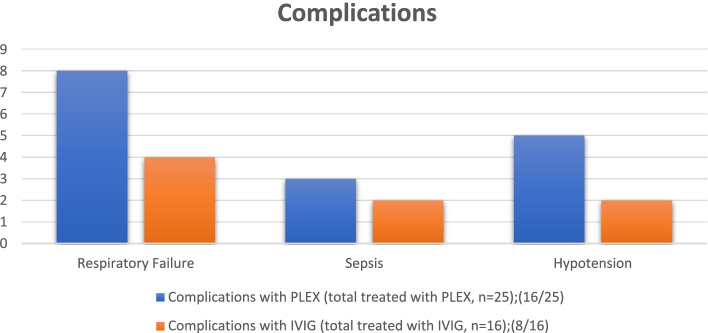
Complications associated with the treatment given to the patients

A total of 25 (50%) patients developed significant complications. Catheter-related complications were more in patients treated with PLEX. One patient died due to respiratory failure. 16 (64%) out of 25 patients treated with PLEX and 8 out of 16 patients treated with IVIG developed complications. 93% of patients with respiratory failure (*n* = 15) had Hughes Score ≥ 3 on admission as compared to 74% of patients without respiratory failure (*n* = 35) (*p* = 0.005). Improvement in score on discharge was 0.15 ± 0.05 in patients with respiratory failure as compared to 0.3 ± 0.11 improvement in score in patients without respiratory failure (*p* = 0.089) (Fig. 4).

## Discussion

Guillain-Barre Syndrome (GBS) is a severe but rare post-infectious immune-mediated neuropathy resulting from autoimmune nerve destruction in the peripheral nervous system. It results in symptoms such as numbness, tingling, weakness, and sometimes paralysis [16]. The age of onset for GBS may range from 0 to 85 years, but the mean age has been reported to be 42.8 years [17]. In line with this age distribution, most of the patients were in the second and fifth decades (26 and 22%, respectively) of their lives. Our study showed a male preponderance with a ratio of 2.3: 1, comparable to the findings of other studies [18, 19]. In contrast to the published Indian literature, which suggests a higher incidence of GBS in the period between June–July and September–October concordant with increased incidence of gastrointestinal and respiratory tract infections [20, 21], there was no significant seasonal variation found in our study.

As mentioned, GBS has been associated with infections, such as upper respiratory infection, which often predates GBS onset by 14 days. The most common antecedent infections identified are *Campylobacter jejuni, Cytomegalovirus, Mycoplasma pneumonia, Epstein-Barr virus, influenza virus,* and Japanese encephalitis virus. 50% of patients in our study had antecedent infections, similar to the previous study [18]. In our study, 88% of patients admitted with antecedent events had a high Hughes Score than the 72% admitted without (*p* = 0.099).

Several different topographic types of GBS have been described, such as MFS, Bickerstaff Brainstem Encephalitis, paraparetic variant, pharyngo-cervico-brachial variant, sensory GBS, ataxic GBS, pandysautonomia, etc. Classic GBS was most seen in our study (94%). With three cases (6%) of atypical GBS, our study is comparable to a study by Bogliun et al. [22], who had 7.25% of atypical GBS variants. Among the three atypical cases, two patients were diagnosed with the paraparetic variant, while one patient had distal paresthesia with bifacial palsy. All the atypical variants had a good prognosis with complete recovery in 4 weeks with conservative management.

Several studies have noted variable involvement of cranial nerves ranging from 50 to 75%. Dhadke et al. [23] had 62.5% of patients with cranial nerves involvement. Bhargava et al. [18] have noted cranial nerve involvement in 49%. In our study, cranial nerve involvement was seen in 34% (*n* = 50) of patients. The facial nerve was the most involved cranial nerve seen in 28% (*n* = 50) of our patients. Furthermore, all the patients with cranial nerve palsies had high Hughes Scores and marked quadriparesis, making them bed-bound, suggesting greater severity of the illness. Cranial nerve involvement was significantly associated with higher Hughes Scores (≥3) on admission (*p* = 0.0087) in 88% of patients compared to 76% of patients without cranial nerve involvement. This is comparable to other studies [18]. Patients without C.N. involvement had significant improvement in scores of 0.38 ± 0.08 in contrast to the patients with cranial nerve involvement (0.13 ± 0.04) (*p* = 0.008).

We used Modified Rajabally’s criteria [14] for classifying GBS using NCV for grouping GBS into its subtypes, such as AIDP, AMAN, and AMSAN. The demyelinating subtype is shown to be more common in western populations [22]. Similar results were seen in our study as well as several others from India [18, 20]. The second most common variant of GBS in the present study was AMAN in 32% of patients. AMAN mostly affects children and young people with abrupt onset of motor weakness associated with transient neck and back stiffness with resolution within a day [24]. AMSAN shares many pathological features with AMAN but differs based on the age of onset (usually adults rather than children), involvement of sensory nerves, course (protracted), and outcome (usually severe residual disability) [25]. About 6% of patients had normal nerve conduction studies. In our study, 82% (*n* = 27) patients with demyelinating subtype had sensory complaints as compared to 15% (*n* = 20) patients with axonal subtype (*p* = 0.009). There is no statistical significance in Hughes Score in patients who presented with axonal subtype compared to patients with demyelinating subtype (*p* = 0.06). Also, there was no significant improvement in score on discharge in patients with axonal subtype than demyelinating subtype (*p* = 0.078).

Albuminocytological dissociation was present in 62% of patients, similar to the previous studies [23]. However, few studies have reported more than 80% of patients with albuminocytological dissociation [18, 22]. An elevated CSF protein with normal cell count may be found on initial (usually within 2 weeks) CSF analysis in 50% of patients and more than 90% of patients at the peak of the disease [24]. Thus, CSF pleocytosis is a significant red flag, which raises the question of infectious (HIV, CMV, Lyme, sarcoid), carcinomatous, or lymphomatous polyradiculoneuropathy [25]. In our study, the presence of albuminocytological dissociation did not impact outcomes in terms of improvement in Hughes score (*p* = 0.77).

Most of the patients in our study were within Hughes’s scale of 4 (38%). That is to say, many patients presented with severe functional disabilities. Further, 50% (*n* = 25) of patients in our study were treated with PLEX. In our study, treatment either with PLEX or IVIG did not significantly differ in short-term functional outcomes regarding improvement in Hughes Score (*p* = 0.89). If administered within the first few weeks of GBS, both PLEX and IVIG are effective therapies for adult and pediatric patients [26]. Along with efficacy, cost-effectiveness is an important factor for treatment choice, especially in developing countries. Similar situations are observed in GBS management, in which the currently approved treatments have shown equal efficacy. In developing countries, using small volumes of PLEX may bring down the cost when compared to IVIG. The main limitations for the use of PLEX would be the availability of technical expertise and support [18].

The mean time to improve power by grade 1 in demyelinating neuropathy (*n* = 22) was 6 ± 1.2 days which was less as reported in the past [22]. Patients who had axonal subtype took almost double-time compared to demyelinating subtype to improve power grade by 1 (10.5 days and 6 days respectively) (*p* = 0.02). The mean time to improve power grade by 1 was lower for 30–60 years age group (7 days), lower for males (6.5 days), more extended if the preceding event was diarrhea (13 days), less with PLEX (8.2 days) than IVIG. However, these differences were not statistically significant (*p* > 0.05). But patients who had cranial nerve involvement took statistically significant time to improve power grade by 1(12 days) as compared to those who did not have cranial nerve involvement (7 days) (*p* < 0.001). The mean duration of hospital stay for our patients was around 15 days for demyelinating neuropathy (*n* = 27), which is less compared to the 18 days duration of hospital stay for axonal neuropathy (*n* = 20) (*p* = 0.03). Many of the confounding factors related to the central line infection and ventilator-associated pneumonia (VAP) may have played a role in the prolonged hospital stay. The mean duration of hospital stay was more for those treated with PLEX (22.8 ± 3.1 days) than those treated with IVIG (15.3 ± 2.1 days) (*p* = 0.01). This finding is similar to an Indian study by Sudulagunta et al. [17] but differs from the Western study Macrondes et al. [27]

## Limitations

Our study is an observational study consisting of patients with GBS only. We did not include control subjects for comparison for outcomes. Our hospital catered to the population with lower socio-economic backgrounds, so our findings might not represent other parts of the country. Being a tertiary care center, most of our patients were referred from different health care centers after primary evaluation, a factor that might be responsible for significant morbidity on admission. In addition, many confounding factors may have played a role in prolonged hospital stay, like central line and VAP complications. Finally, as a developing country, several factors were involved in choosing between PLEX and IVIG as a treatment in our study, financial restrictions being one of the most common. Despite the limitations, this study gives detailed information on a unique population suffering from GBS. This study will serve as a baseline for further studies with larger sample size and matched case-control studies.

## Conclusions

Early cranial nerve and respiratory involvement in patients presenting with GBS are associated with poor outcomes warranting immediate critical care involvement. In our study, amongst all the subtypes, axonal had poor clinical outcomes. It is imperative to recognize these symptoms, identify the subtype of GBS, and rapidly implement treatment. The mean time to improve power grade by 1 point (Hughes Scale) was double in patients with axonal subtype and significantly higher with cranial nerve involvement. Duration of hospital stay was longer with demyelinating neuropathy and in patients treated with PLEX. Both PLEX and IVIG had similar functional outcomes. Given the variability of infection, disease, and triggers in different populations, further prospective clinical trials with a well-defined population will help us evaluate the impact of different treatment modalities on this disease.

## Data Availability

The datasets generated and/or analyzed during the current study are not publicly available due to patient confidentiality but are available from the corresponding author on reasonable request.

## Abbreviations

GBS: Guillain- Barre Syndrome
AIDP: Acute Inflammatory Demyelinating Polyneuropathy
AMAN: Acute Motor Axonal Neuropathy
CMAP: Compound Muscle Action Potential
CN: Cranial Nerves
ICU: Intensive Care Unit
CSF: Cerebrospinal Fluid
MFS: Miller Fisher Syndrome
NCV: Nerve Conduction Velocity
AMSAN: Acute Motor and Sensory Axonal Neuropathy
PLEX: Plasma Exchange
IVIG: Intravenous Immunoglobulins
SNAP: Sensory Nerve Action Potential
VAP: Ventilator-Associated Pneumonia
